# Neuromodulation accompanying focused ultrasound-induced blood-brain barrier opening

**DOI:** 10.1038/srep15477

**Published:** 2015-10-22

**Authors:** Po-Chun Chu, Hao-Li Liu, Hsin-Yi Lai, Chung-Yin Lin, Hong-Chieh Tsai, Yu-Cheng Pei

**Affiliations:** 1 Department of Electrical Engineering, Chang Gung University, 259 Wen-Hwa 1st Road, Kwei-Shan Tao-Yuan, Taiwan, 333; 2 Medical Imaging Research Center, Institute for Radiological Research, Chang Gung University/Chang Gung Memorial Hospital, Taoyuan, Taiwan 333; 3 Department of Physical Medicine and Rehabilitation, Chang Gung Memorial Hospital, 5 Fu-shin Street, Kwei-Shan, Tao-Yuan, Taiwan, 333, R.O.C; 4 School of Medicine, Chang Gung University, 259 Wen-Hwa 1st Road, Kwei-Shan Tao-Yuan, Taiwan, 333, R.O.C; 5 Department of Neurosurgery, Chang Gung Memorial Hospital, 5 Fu-shin Street, Kwei-Shan, Tao-Yuan, Taiwan, 333, R.O.C

## Abstract

Burst-mode focused ultrasound (FUS) induces microbubble cavitation in the vasculature and temporarily disrupts the blood-brain barrier (BBB) to enable therapeutic agent delivery. However, it remains unclear whether FUS-induced BBB opening is accompanied by neuromodulation. Here we characterized the functional effects of FUS-induced BBB opening by measuring changes in somatosensory evoked potentials (SSEPs) and blood-oxygen-level dependent (BOLD) responses. Rats underwent burst-mode FUS (mechanical index (MI) of 0.3, 0.55 or 0.8) to the forelimb region in the left primary somatosensory cortex to induce BBB opening. Longitudinal measurements were followed for up to 1 week to characterize the temporal dynamics of neuromodulation. We observed that 0.8-MI FUS profoundly suppressed SSEP amplitude and prolonged latency, and this effect lasted 7 days. 0.55-MI FUS resulted in minimal and short-term suppression of SSEP for less than 60 minutes and didn’t affect latency. BOLD responses were also suppressed in an MI-dependent manner, mirroring the effect on SSEPs. Furthermore, repetitive delivery of 0.55-MI FUS every 3 days elicited no accumulative effects on SSEPs or tissue integrity. This is the first evidence that FUS-induced BBB opening is accompanied by reversible changes in neuron responses, and may provide valuable insight toward the development of FUS-induced BBB opening for clinical applications.

Focused ultrasound (FUS) with the presence of circulating microbubbles can temporarily disrupt the blood-brain barrier (BBB)^1^. FUS induces microbubble cavitation in the vasculature, and the resultant shear stress temporarily disrupts tight junctions to enhance BBB permeability^2^. FUS-induced BBB opening can aid in the delivery of therapeutic agents to enhance the treatment of diseases such as brain tumours^3^,^4^. FUS has been applied in large animals and non-human primates, and it has the advantage of non-invasiveness, spatial accuracy and reversibility if delivered at a proper intensity^5^,^6^. Indeed, its clinical potential is emerging as it offers a unique opportunity to deliver drugs to a localized brain area for patients with neurological disorders, an approach that can enhance the therapeutic effect and decrease whole-brain influence^4^.

However, clinical implementation of FUS requires careful evaluation of its safety and efficacy^6^,^7^. For a novel therapy such as FUS-induced BBB opening, safety issues continue to be a concern and research is needed to understand at what point the risks outweigh the benefit. For example, the intensity that opens the BBB without injuring the brain remains to be determined^8^,^9^,^10^,^11^. Also, it remains unclear whether FUS-induced BBB opening also induces neuromodulation. Given that FUS-induced BBB disruption might be applied in patients in the near future, it is mandatory to assess its safety and effect on neuronal functions.

FUS without microbubbles (FUS-alone) has been reported to induce neuromodulation simply through ultrasound-induced mechanical stress. For example, FUS-alone suppresses epileptic activity^12^, decreases GABA levels in cerebral spinal fluid^13^, and enhances evoked motor behaviours^14^ and human tactile acuity^15^. Furthermore, FUS-alone enhances neuronal activity in the motor cortex, but suppresses activity in the visual cortex^16^, indicating that the effects of FUS differ across cortical areas. However, the exposure condition of FUS used for BBB opening is different from that used in the aforementioned FUS-induced neuromodulation, so it is important to assess whether FUS-induced BBB opening is accompanied by brain modulation.

In this study, we measured somatosensory evoked potentials (SSEPs) and blood-oxygen-level-dependent (BOLD) responses to assay neuromodulation that might accompany FUS-induced BBB opening. Stimulus-driven SSEPs^17^,^18^ and BOLD responses^19^,^20^ are widely used to measure cortical neuromodulation^16^,^21^. We used SSEPs because the latency and amplitude of the first maximum voltage (P1) after stimulus onset reflect activity in the primary somatosensory cortex driven by thalamic input^22^,^23^. Additionally, we took advantage of the whole-brain scale of BOLD responses to locate FUS-induced neuromodulation^19^,^20^. Measurements were performed at multiple time points to observe the temporal dynamics of neuromodulation. Furthermore, histology was performed to characterize the changes at the tissue level. Finally, repetitive FUS-induced BBB opening was also performed to evaluate accumulative functional and histological effects.

## Results

### FUS-induced BBB opening

**Figure 1:**
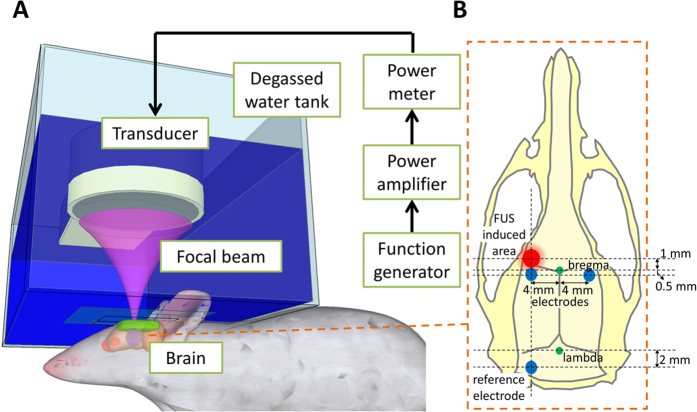
Schematic drawing to illustrate FUS delivery and the placement of epidural electrodes. (**A**) FUS delivery set-up. (**B**) The focal zone of FUS (red) and the position of epidural electrodes (blue) on the skull. Green circles denote the bregma and lambda.

Animals were placed in the prone position to receive FUS through a water tank (Fig. 1A). Immediately after intravenous injection of SF6-coated microbubbles, burst-tone-mode ultrasound was delivered at the centre of the focal zone located 1–2 mm under the skull, overlying the left primary somatosensory cortex forelimb region (S1FL) (Fig. 1B). To localize the spatial distribution of FUS-induced BBB opening, Evans blue dye was injected intravenously immediately following FUS.

To examine neuronal activity under various parameters, we used FUS (frequency = 400 kHz) with a mechanical index (MI) of 0.3, 0.55 or 0.8, covering a spectrum of known biological and pathological effects that range from no BBB opening to BBB opening accompanied by possible vascular insults. Specifically, the 0.3-MI FUS is lower than the threshold that induces BBB opening (MI = 0.46)^24^, 0.55-MI FUS is considered within the safe range, and 0.8-MI FUS induces BBB opening and red blood cell (RBC) extravasation^9^,^11^. Two more control groups were included: the first control group received microbubbles without FUS (denoted as control); the second control group received 0.8-MI FUS for 120 s without the presence of microbubbles (0.8-MI FUS-alone). This group was included to determine whether neuromodulation was induced by ultrasound alone and whether it was accompanied by BBB opening.

**Figure 2:**
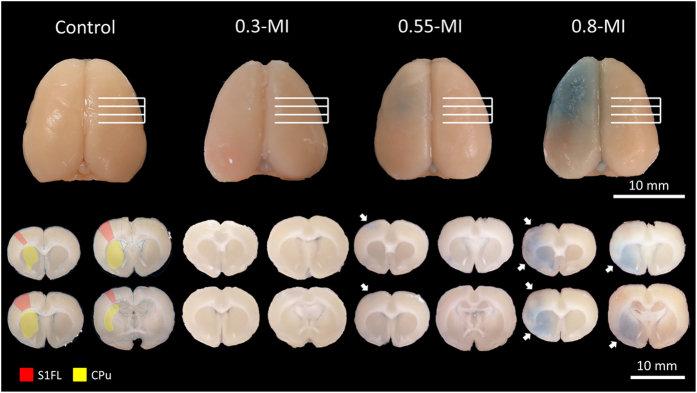
Representative gross views and brain slices showing regions with BBB-opening, as evidenced by Evans blue dye leakage. Leakage was found in the left S1FL for both the 0.55- and 0.8-MI groups, but not for the control or 0.3-MI groups. The red and yellow regions denote left S1FL and caudate putamen (CPu), respectively. The white arrows indicate regions with Evans blue dye leakage.

Evans blue dye leakage revealed FUS-induced local BBB opening in the left S1FL for the 0.55- and 0.8-MI groups, but not for the control or 0.3-MI groups (Fig. 2). Furthermore, 0.8-MI FUS induced more widespread and intense Evans blue dye leakage than 0.55-MI FUS. Finally, 0.8-MI FUS-alone did not induce BBB opening (see Supplementary Fig. S1A online).

### Neuromodulation identified from SSEP changes

**Figure 3:**
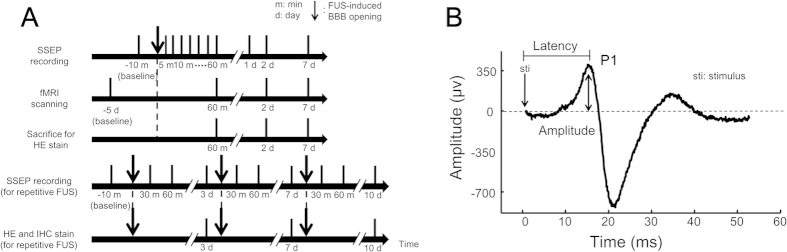
The experimental design. (**A**) Time course of experiments. (**B**) Example SSEP waveform measured from left S1FL, elicited by right forepaw electrical stimulation.

The SSEPs were first measured at baseline (10 min before FUS). The immediate effects of FUS were then investigated by frequent measurements during the first hour (5, 10, 20, 30, 40, 50 and 60 min post-FUS), and long-term effects by follow-up measurements on days 2 and 7 (Fig. 3A). The SSEPs were elicited by forepaw electrical stimulation and were recorded by the epidural electrodes placed proximal to S1FL (Fig. 1B). The amplitude and latency of the peak of the first positive component (denoted as P1) that occurred 13–18 ms after stimulus onset were measured (Fig. 3B). The change in SSEPs was defined as SSEP—baseline SSEP divided by baseline SSEP. This normalization was adopted because the baseline SSEPs varied substantially across animals.

**Figure 4:**
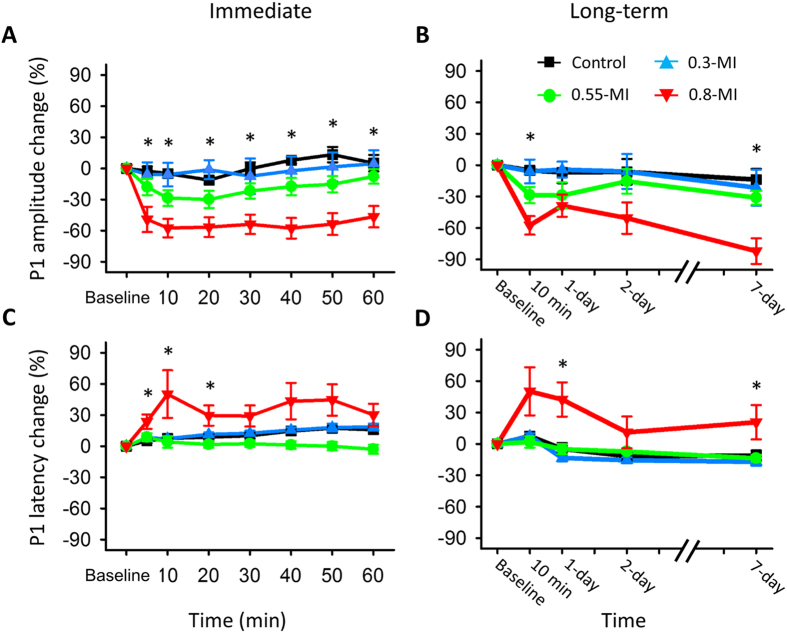
The immediate and long-term changes of SSEP in the left S1FL. (**A**) Change in P1 amplitude within the first hour post-FUS. P1 amplitude was reduced for the 0.55 and 0.8-MI groups, with a stronger reduction for the latter. (**B**) Change of P1 amplitude on long-term follow-up. P1 was persistently reduced for the 0.8-MI group, transiently reduced for the 0.55-MI group, and unaffected for the control or 0.3-MI groups. (**C**) Change in P1 latency within the first hour post-FUS. P1 latency was prolonged for the 0.8-MI group, but not for the other groups. (**D**) Change in P1 latency on long-term follow-up. P1 latency was markedly prolonged for the 0.8-MI group. *denotes significant group differences.

Within the first hour, FUS reduced the P1 amplitude for the 0.55- and 0.8-MI groups, but not for the control or 0.3-MI groups (Fig. 4A), suggesting that the reduction in SSEPs only occurred when the BBB was opened. Repeated-measures ANOVA indicated that the magnitude of the reduction differed among the four groups (group effect, *F* (3, 28) = 10.84, *p* < 0.001; interaction effect, *F* (18,168) = 0.379, *p* = 0.99). The difference in P1 amplitude changes among the three groups was observed 5 min post-FUS *(F* (3, 31) = 4.32, *p* < 0.05) and lasted for the remainder of the experiment (all *p* < 0.05). Post-hoc analysis showed that 0.8-MI FUS reduced P1 amplitude at all time points (*p* < 0.05), while 0.55-MI FUS reduced P1 amplitude 10 min post-FUS at borderline significance level (*p* = 0.05). Interestingly, while the P1 amplitude had not recovered to the baseline level (*p* < 0.05) 60 min post-FUS for the 0.8-MI group, changes in P1 amplitude were transient for the 0.55-MI group.

Follow-up 2 and 7 days later showed that P1 amplitude was reduced for the 0.8-MI group, but not for the other three groups (Fig. 4B) (group effect, *F* (3, 24) = 10, *p* < 0.001; interaction effect, *F* (9, 72) = 0.32, *p* = 0.97). Post-hoc analysis at each time point showed that only 0.8-MI FUS reduced the P1 amplitude (*p* < 0.05). Furthermore, the P1 amplitude in the 0.8-MI group had not recovered to the baseline level (*p* < 0.01) 7 days post-FUS. By contrast, the P1 amplitude for the 0.55-MI group recovered to the baseline 1 day post-FUS, indicating that neuromodulation induced by 0.55-MI FUS was reversible.

Within the first hour, P1 latency was prolonged for the 0.8-MI group, but not for the other three groups (Fig. 4C) (group effect, *F* (3, 28) = 4.54, *p* < 0.01; interaction effect, *F* (18, 168) = 1.55, *p* = 0.12). Specifically, the 0.8-MI group had the highest P1 latency 20–60 min post-FUS (post-hoc analysis, *p* < 0.05). Furthermore, P1 latency had not recovered to baseline level 60 min post-FUS for the 0.8-MI group (*p* < 0.05), as was the case with amplitude.

On long-term follow-up, the effect on P1 latency was still observed for the 0.8-MI group, but not for the other three groups (Fig. 4D) (group effect, *F* (3, 26) = 10.51, *p* < 0.001; interaction effect, *F* (9, 78) = 0.36, *p* = 0.95). Specifically, the 0.8-MI group had the highest P1 latency 1 and 7 days post-FUS (post-hoc analysis, *p* < 0.05).

FUS did not alter P1 amplitude or latency on the non-FUS (right) side (see Supplementary Fig. S2 online), indicating that the neuromodulation effect was localized. Furthermore, 0.8-MI FUS-alone induced no changes in SSEP amplitude or latency (see Supplementary Fig. S1B online), again demonstrating that neuromodulation was related to the occurrence of BBB opening, but not FUS alone.

### Neuromodulation identified from BOLD response change in functional MRI (fMRI)

MRI was performed using a 7 Tesla Bruker BioSpec system (Bruker Corp., Billerica, MA, USA). fMRI scanning was performed at baseline, 1 h, 2 days and 7 days after FUS. BOLD responses elicited by forepaw electrical stimulation were analyzed and regions of interest (ROIs) in S1FL were applied to the co-registered data for statistical analysis.

**Figure 5:**
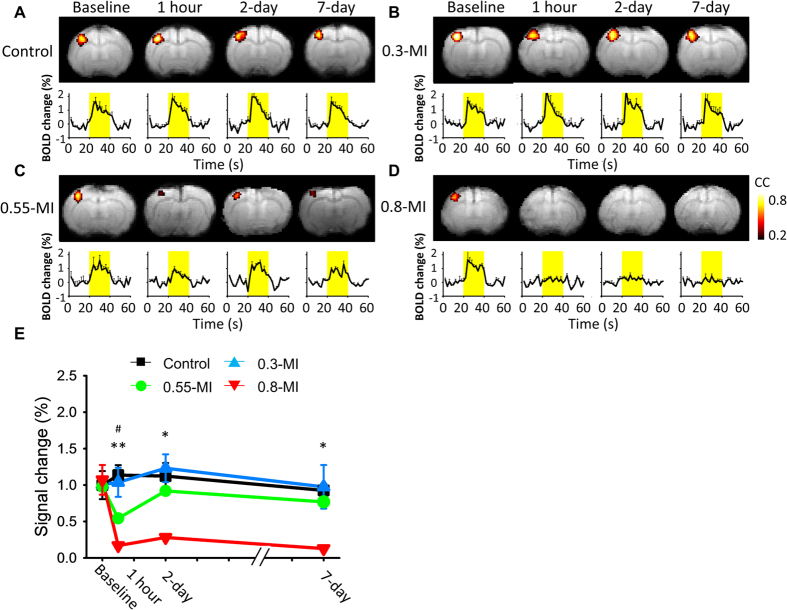
Spread and magnitude of the BOLD responses. (**A**) Control group. Yellow bars indicate the stimulation periods. (**B**) 0.3-MI group. (**C**) 0.55-MI group. (**D**) 0.8-MI group. (**E**) Time course of the effects on BOLD responses. The neuromodulation effect was reversible for the 0.55-MI group, but not for the 0.8-MI group. ^#^denotes a significant difference between the 0.55-MI and control groups. *denotes a significant difference between the 0.8-MI and control groups.

BOLD responses were reduced when tested 1 h and 2 days after FUS for the 0.8-MI group, and the effect had dissipated after 2 days for the 0.55-MI group, and was not observed at all for the control or 0.3-MI groups (Fig. 5). The magnitude of the reduction differed significantly across the four groups (group effect, *F* (3, 12) = 15.35, *p* < 0.001; interaction effect, *F* (9, 36) = 3.046, *p* < 0.01). Post-hoc analysis indicated that 0.8-MI FUS reduced BOLD responses at all time points (*p* < 0.05), while 0.55-MI FUS reduced BOLD responses 1 h post-FUS. Furthermore, the 0.8-MI group induced a higher magnitude of FUS-induced reduction in BOLD responses than did the 0.55-MI group when tested 1 h post-FUS.

BOLD responses for the 0.55-MI group had recovered to baseline levels 2 days post-FUS (*p* = 0.71), as was the case with SSEPs. However, the reduction in BOLD responses was more robust than that in SSEPs 1 h post-FUS (*F* (1, 18) = 12, *p* < 0.01) when these effects were normalized to their respective baseline levels. By contrast, the reductions observed in SSEPs and BOLD responses were comparable 2 and 7 days post-FUS (*p* > 0.05, respectively). The 0.8-MI FUS-alone did not suppress the BOLD responses, as was found with SSEPs (see Supplementary Fig. S1C online).

### Histological examinations

**Figure 6:**
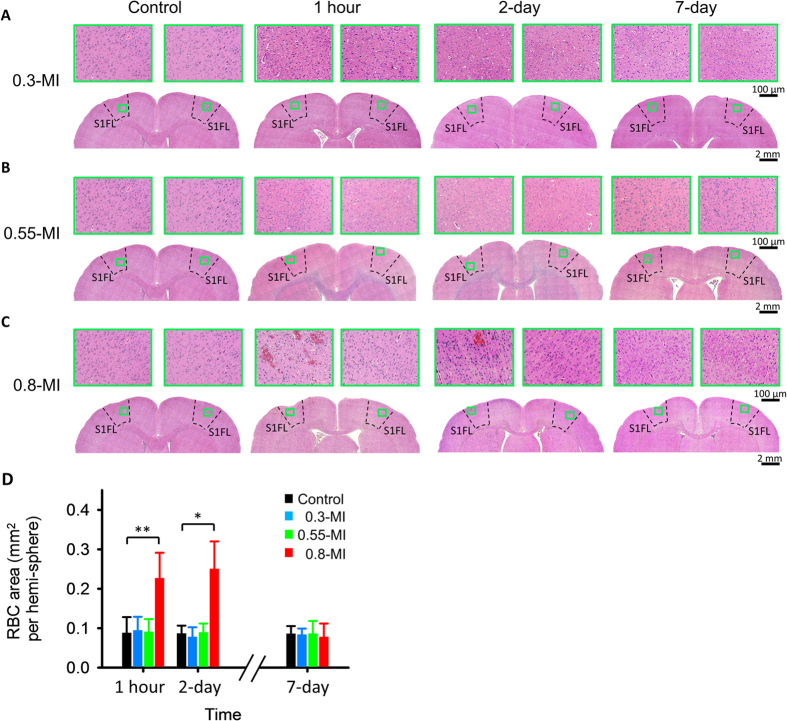
Example HE-stained slices 1 h, 2 days and 7 days post-FUS, and RBC area for the three groups. (**A**) 0.3-MI and control groups, whole brain: 40 X; detailed tissue: 200 X. (**B**) 0.55-MI and control groups. (**C**) 0.8-MI and control groups. (**D**) RBC area per hemisphere. RBC area was largest for the 0.8-MI group 1 h and 2 days post-FUS. *p < 0.05; **p < 0.01.

Histological changes induced by FUS were evaluated at multiple time points (1 hour, 2 days and 7 days post-FUS) (Fig. 6). The four groups exhibited differences in the area occupied by RBC 1 h *(F* (3, 23) = 11.53, *p* < 0.01) and 2 days (*F* (3, 23) = 4.55, *p* < 0.05) post-FUS, but there was no difference 7 days post-FUS *(F* (3, 23) = 0.11, *p* = 0.96). Post-hoc analysis showed that RBC extravasations were highest for the 0.8-MI group 1 h (*p* < 0.01) and 2 days (*p* < 0.05) post-FUS. Finally, 0.8-MI FUS-alone induced no RBC extravasations (see Supplementary Fig. S1D online).

### The effect of repetitive FUS

**Figure 7:**
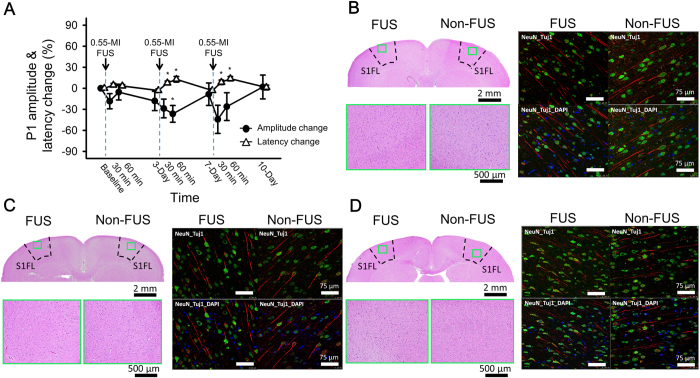
SSEPs and representative histological and immunohistochemistry (IHC) examinations with the repetitive 0.55-MI FUS-induced BBB opening. (**A**) Changes in P1 amplitudes and latencies in measured SSEPs throughout the repetitive FUS treatment course. (**B–D**) HE as well as NeuN- and TuJ1-stained IHC was performed with animals sacrificed 3-days after the first (in **B**), second (in **C**), and third (in **D**) FUS-induced BBB opening. *indicates *p* < 0.05.

To evaluate whether repetitive 0.55-MI FUS, which is analogous to multiple rounds of drug delivery for clinical application, induced accumulated functional or morphological changes in the brain, we conducted FUS three times over a three-day interval and evaluated the SSEPs and performed histological/immunohistochemistry (IHC). Immediately after each FUS, the P1 amplitude was transiently reduced and the P1 latency was transiently prolonged, and both P1 amplitude and latency recovered during the long-term follow-up. Importantly, repetitive FUS showed no accumulated effect; the P1 amplitude fully recovered to its baseline value in the follow-up examinations (Fig. 7A) ((F (3, 30) = 0.728, p = 0.543), for P1 amplitude at baseline, and 3, 7, and 10 days follow-up). Again, the P1 latency also fully recovered in the follow-up examinations (Fig. 7A) ((F (3, 30) = 1.354, *p* = 0.276), for P1 latency at baseline, and 3, 7, and 10 days of follow-up).

Histological and IHC examinations were evaluated for animals with 4, 7 and 10 days follow-up, corresponding to those with 1, 2, and 3 FUS dosages, respectively (Fig. 7B–I). First, HE stains demonstrated that there were not any RBC extravasations for any of the time points. NeuN stains that are specialized for imaging neuronal nucleus showed mature cells functionally and morphologically at all of the time points. TuJ1 stains that are specialized for neuron-specific beta-III tubulin showed no changes in neuronal differentiation at any time point. To sum up, the functional integrity was unaltered by repeated FUS.

## Discussion

In this study, we demonstrated neuromodulation accompanying FUS-induced BBB opening, as evidenced by changes in SSEPs and BOLD responses following FUS. This effect was transient, lasting for less than 60 min following 0.55-MI FUS, but lasted at least 7 days following 0.8-MI FUS. Robust reduction in SSEP and BOLD amplitudes was observed on the FUS-exposed side, but not on the non-FUS side, indicating that neuromodulation occurred mainly in the focal zone of FUS. We also showed that repetitive 0.55-MI FUS induced no accumulated effects on SSEP or tissue neuron integrity, indicating that repetitive use of FUS-induced BBB opening can be clinically safe. The relatively narrow safety window and robust neuromodulation observed in this study highlights the importance of understanding the physiological effect of FUS-induced BBB opening before it can be applied in patients with neurological disorders.

Several mechanisms could account for the neuromodulation accompanying FUS-induced BBB opening. FUS may reduce the efficiency of synaptic projections from the thalamus to S1FL, or suppress intracortical processing in the S1FL region. Evans blue staining indicated that the ultrasound could be focused on S1FL while avoiding the thalamus, suggesting that suppression occurred predominantly in the S1FL. However, the mechanism whereby FUS-induced cavitation affects neuronal activity remains unclear. We have three hypotheses that need to be tested in future experiments. First, the mechanical force of cavitation may influence the transporters involved in neuronal communication, resulting in the suppression of postsynaptic potentials and action potentials. This mechanism is analogous to a brain concussion^25^,^26^, the mildest form of traumatic brain injury^27^. Second, the mechanical force may transiently block axonal conduction by affecting action potential propagation^28^. Finally, the capillaries may narrow following BBB opening^29^, resulting in reduced blood flow and oxygen/glucose delivery and suppression of neuronal activity.

The dramatic differences in brain modulation observed between the 0.55- and 0.8-MI groups may be due to critical differences in the biological effects of these stimuli. One possibility is that 0.8-MI FUS induced RBC extravasations, while 0.55-MI FUS did not^9^,^11^. High MI exposures have been shown to cause RBC extravasations accompanied by transient capillary contraction^30^, resulting in transient ischemia and suppression of cortical function^31^,^32^. Another possibility is that haemoglobin in the extravasated RBCs can act as a neurotoxin that transiently suppresses neuronal function^33^,^34^.

While, to our knowledge, neuromodulation accompanied by FUS-induced BBB opening has never been previously demonstrated, the effect of FUS-alone without microbubbles has been widely reported. For example, Min *et al.* first found that FUS-alone to the thalamus reduced electroencephalographic activity^12^, while Yang *et al.* reported that FUS-alone decreased GABA levels in cerebrospinal fluid^13^. Tufail *et al.* found that FUS-alone stimulated neuronal activity, increased synchronous oscillations and evoked motor behaviours^14^. Furthermore, King *et al.* reported behavioural correlates of FUS-induced neuromodulation as reflected by enhanced electromyography activity^35^, while Yoo *et al.* found that 0.5-MI FUS to the visual cortex suppressed visual evoked potentials^16^. However, in the present study we observed no neuromodulation for the 0.8-MI FUS-alone group. The discrepancy may be due to the fact that our pulse repetition frequency was much lower than that used in the previous studies and thus, may have been insufficient to induce neuromodulation.

Non-invasive neuromodulation has major clinical implications^36^. Available brain modulation methods such as repetitive transcranial magnetic stimulation (rTMS)^37^,^38^ and transcranial direct current stimulation (tDCS)^39^,^40^ are limited by their spatial resolution. Because of the inductive nature of magnetic stimulation, the area of modulation induced by rTMS is rather wide (on the order of several centimetres), and limited to the cortical surface^37^. The current of tDCS passes through the brain between the two electrode sites so that the effect of neuronal polarization is also widespread^41^. In this study, we showed that robust and reversible non-invasive neuromodulation could be achieved by choosing appropriate FUS parameters. FUS-induced neuromodulation can be focused on a specific brain area^15^,^16^ and can reach deep brain structures^42^.

The P1 component of SSEPs^43^ originates from sensory neurons that receive thalamocortical projections^44^,^45^. As such, the activity of the early processing stages is reflected in both the latency and amplitude of P1^22^,^23^. Therefore, the decrease in P1 amplitude and the prolongation of P1 latency reflect suppression in the early cortical processing stage. We also noted that FUS suppressed the first minimum voltage (N1) after the stimulation, which reflects the slow, long latency sink in layers I/II^46^ and is generated by excitatory cortical events^47^. Future studies are needed to analyze the effect of FUS on intracortical processing using other electrophysiological measurements.

We used BOLD responses to confirm that neuromodulation occurred in the ROI, exploiting the high-spatial resolution of fMRI^19^,^20^. It is interesting that the decrease in BOLD responses was disproportionally higher than that of SSEPs, perhaps due to the narrowing of capillaries induced by BBB opening^29^. In this study, SSEPs and BOLD responses showed a parallel temporal pattern of suppression, thus providing cross-validation of the observed neuromodulation.

Histological examination showed that BBB opening induced by 0.8-MI FUS was accompanied by RBC extravasations, which paralleled the suppression of SSEPs and BOLD responses. Indeed, extravascular RBCs are commonly used to measure the degree of brain insult^48^. Notably, the magnitude of suppression induced by 0.8-MI FUS was higher for BOLD responses than for SSEPs. Specifically, a nearly total elimination of BOLD responses was observed compared with only a partial decrease in SSEPs, implying that the change in BOLD responses reflects both suppression of neural activity and a change in vascular properties^19^,^49^. By contrast, 0.55-MI FUS-induced BBB opening did not induce any RBC extravasations compared with controls, indicating that BBB opening parameters were within the safe range. Most importantly, 0.55-MI FUS caused a substantial reduction in neuronal activity and might thus be a suitable intensity to achieve neuromodulation.

FUS-induced BBB opening has potential for clinical application^2^,^7^,^50^, and repetitive FUS might be needed for drug delivery to the brain. For example, chemotherapy for brain tumors is commonly delivered once or twice per week, so the biological effect of repetitive FUS determinates its feasibility and safety. However, to the best of our knowledge, neuromodulation accompanying multiple FUS-induced BBB openings has not been evaluated. This study showed that repetitive FUS over an interval of 3 days might be considered safe. Further studies are needed to evaluate the range of repetitive FUS parameters that show satisfactory drug delivery while avoiding accumulated neuromodulation.

## Methods

### FUS instrumentation and calibration

The FUS instrument consisted of a function generator (33120A; Agilent, Palo Alto, CA, USA), a radiofrequency power amplifier (150A100B; Amplifier Research, Souderton, PA, USA) and a focused ultrasound transducer (IMASONIC, Besançon, France; diameter = 60 mm, radius of curvature = 80 mm, frequency = 400 kHz and electric-to-acoustic efficiency = 70%) (Fig. 1A). A FUS frequency of 400 kHz was used because it can penetrate the skull^51^. The diameter and length of the half-maximum pressure amplitude of the ultrasound field were 2 and 15 mm, respectively, when measured in a free field within an acrylic tank filled with deionized/degassed water. The acoustic pressure was measured from a needle type hydrophone. An *ex vivo* rat skull was placed between the transducer and hydrophone to acquire the attenuated acoustic pressure.

### Animal experimental design

The methods were carried out in accordance with the approved guidelines for the Care and Use of Laboratory Animals (NIH publication no. 86–23, revised 1987). All experimental protocols were approved by the Institutional Animal Care and Use Committee of Chang Gung Memorial Hospital. A total of 118 animals (male Sprague-Dawley rats, 250–300 g) were used: *N* = 44 for the SSEP experiment, *N* = 20 for the fMRI experiment and *N* = 54 for the histology experiment. To examine neuronal activity under various parameters, we used FUS powers of 0.56, 1.13 and 1.98 W, equivalent to acoustic pressures of 0.2, 0.35 and 0.5 MPa or MI of 0.3, 0.55 and 0.8, respectively. The MI is defined as the peak negative pressure divided by the square root of frequency.

For all experiments, animals were divided into six groups. The first control group received microbubbles without FUS (denoted as control). The second control group received 0.8-MI FUS for 120 s without the presence of microbubbles (0.8-MI FUS-alone). This group was included to determine whether neuromodulation is induced by ultrasound alone and whether it is accompanied by BBB opening (see Supplementary Information online). The three experimental groups received microbubbles and FUS of 0.3, 0.55 or 0.8 MI for 120 s; the control, 0.3-, 0.55- and 0.8-MI groups thus received different ultrasound intensities.

In the repetitive 0.55-MI FUS group, FUS of 0.55 MI with microbubbles was delivered 3 times over a 3-day interval and SSEP was recorded at multiple time points (immediately before each FUS, and 30 and 60 minutes after each FUS and 3 days after the last FUS). Furthermore, additional animals were scarified immediately after the first, second, and third FUS for histological and IHC examinations.

For SSEP experiments, eight animals were included in each of the control, 0.3-, 0.55- and 0.8-MI groups, and six animals were included in the 0.8-MI FUS-alone and repetitive 0.55-MI groups. For fMRI evaluation, four animal experiments were conducted in each group. For histological evaluation, nine animals were used in each group (see Supplementary Table S1 online).

### FUS-induced BBB opening

Rats were initially anesthetized with 3% isoflurane in 21% O_2_ and 79% N_2_. A cannula was inserted into the tail vein for intravenous administration of Dexdomitor® (Dexmedetomidine; Orion, Espoo, Finland) at 0.1 mg/kg to maintain anesthesia after isoflurane was discontinued. The scalp overlying the FUS area was removed for the SSEP and histology experiments. Animals were placed in the prone position directly under an acrylic water tank that contained a 4 × 4 cm^2^ window sealed with a thin film of polyethylene membrane to allow the ultrasound to penetrate through its base (Fig. 1A). The space between the skull and the thin-film window was filled with ultrasound gel. Lipid-shell Sulfur hexafluoride (SF6)-encapsulated microbubbles (SonoVue®, Bracco Diagnostics Inc., Milan, Italy; 2–5 μm in mean diameter, 0.1 mL/kg) and heparin (0.03 ml/kg; Agglutex, China Chemical and Pharmaceutical Corporation, Taipei, Taiwan) were administered intravenously after dilution with normal saline solution to a total volume of 0.3 ml. The biological effects induced by these dosages of microbubbles and FUS intensities have previously been established^3^,^9^,^52^,^53^. At this microbubble dosage, BBB opening is expected to occur when the ultrasound intensity is above 0.5 MI. BBB opening without RBC extravasations has been observed at a comparable ultrasound intensity and microbubble dosage, while RBC extravasations occur when the microbubble dosage is doubled^54^,^55^, indicating that the biological effects of FUS are dependent on both ultrasound intensity and microbubble dosage. Immediately after injection of microbubbles, burst-tone-mode ultrasound was delivered at the centre of the focal zone located 1–2 mm under the skull, overlying the left primary somatosensory cortex forelimb region (S1FL) (coordinates from Bregma: AP, + 1 mm; ML, −4 mm; Fig. 1B). The burst length was 10 ms and the pulse-repetition frequency was 1 Hz. To localize the spatial distribution of FUS-induced BBB opening, Evans blue dye (3% in saline, 1 mL/kg) was injected intravenously immediately following FUS for all experiments, except for the fMRI experiment.

### SSEP measurements

Epidural electrodes were implanted 2 days before FUS. To perform this surgical procedure, animals were anesthetized with 0.1 mg/kg Dexdomitor^®^ by subcutaneous injection (once per 30 min). Animals were immobilized on a stereotaxic apparatus (Model 900; David Kopf, Tujunga, CA, USA). The electrode sites were 0.5 mm posterior and 4 mm lateral to the bregma to prevent attenuation of FUS, which was centred 1 mm anterior and 4 mm lateral to bregma (Fig. 1B). A reference epidural electrode was positioned 2 mm posterior and 4 mm left-lateral to lambda. These electrodes were attached to the bone using dental acrylic (Type 1 Class 1; Hygenic Corp., Akron, OH, USA). In SSEP experiments, the scalp was replaced by dental acrylic after FUS for all SSEP groups for minimizing the overgrowth of granulation tissue, fixating the electrodes and providing a platform for the connector^56^,^57^. Specifically for repetitive 0.55-MI exposures, scalp clips were employed to temporally close the scalp wound after each FUS, with ampicillin (4 mg/kg) intraperitoneally injected for infection prevention.

A pair of stainless needle electrodes were inserted 1.5 mm apart under the skin of each forepaw for electrical stimulation. For each time point, SSEPs were first measured for stimulation of the right forepaw and then the left forepaw. Electrical stimulation was applied with a stimulator (model DS3; Digitimer Ltd., Welwyn Garden City, UK) triggered by a function generator (Master-9; A.M.P.I, Jerusalem, Israel) with a biphasic square-wave at a constant current of 6 mA, pulse-width of 0.2 ms and frequency of 3 Hz. The SSEPs were recorded for 20 s, sampled at 1 kHz and band-pass filtered between 0.35 and 500 Hz using a multichannel acquisition system (Cerebus; Blackrock microsystems, Salt Lake City, UT, USA). The SSEPs were analysed offline using Matlab (Matlab; Mathworks Inc., Natick, MA, USA).

### fMRI for BOLD signal measurements

Rats were anesthetized with 0.1 mg/kg Dexdomitor^®^ subcutaneously (once per 30 min) after induction with 2–3% isoflurane in 20% O_2_, 75% N_2_ and 5% CO_2_. The water temperature of the circulating pad was adjusted to maintain body temperature at 37 °C. A pressure sensor (SA Instruments, Inc., New York, NY, USA) was positioned under the abdomen of the animal to monitor respiration, which was maintained between 45–55 breaths per min. The receive-only coil (T7399V3; Bruker Corp., Billerica, MA, USA) was placed directly over the head. Magnetic field homogeneity was optimized using standard FASTMAP shimming with first order shims on an isotropic voxel of 7 × 7 × 7 mm^3^ encompassing the imaging slices.

We obtained anatomical images using RARE T2-weighted imaging (TR/TE = 2500/33 ms, FOV = 2.5 × 2.5 cm, slice thickness = 1 mm, and matrix = 156 × 156) for anatomical localization. For functional scans, a gradient-echo echo-planar imaging sequence was used (spectral width = 200 kHz, TR/TE = 2000/20 ms, FOV = 2.5 × 2.5 cm, slice thickness = 1 mm, and matrix = 80 × 80). fMRI scans were acquired for 60 s (30 repetitions), during which stimulation was applied in OFF-ON-OFF blocks followed by a 3-min inter-scan resting period. The period of each OFF/ON block was 20 s. Forepaw electrical stimulation was identical to that used in the SSEP experiment except that the frequency was 12 Hz. Three to five repeated scans were performed to improve measurement precision and to optimize the signal-to-noise ratio.

Image analysis was performed using a custom written program^58^. Images were automatically co-registered and data were averaged across animals to provide group-averaged fMRI maps using a correlation coefficient method with reference to the stimulation paradigm. Bonferroni correction was applied to adjust for multiple comparisons of fMRI maps by dividing the significance level (*p* < 0.05) by the number of brain voxels. ROIs in S1FL were defined on an atlas^59^ and then applied to the co-registered data. The time-course of BOLD responses was calculated for each ROI using the first 10 frames to establish the baseline.

### Histological and Immunohistochemistry (IHC) examinations

Each animal was deeply anesthetized with chloral hydrate (350 mg/kg) and the brain was fixed in 4% buffered neutral formalin. After fixation, the brain was cut into a series of coronal blocks and embedded in paraffin. The blocks were serially sectioned (6 μm thick) and stained with Haematoxylin and Eosin (HE). To quantify the level of brain insults, an Axio Imager microscope (Carl Zeiss, Oberkochen, Germany) was used to collect histological images, and the area of RBCs in the sonicated brain region was automatically quantified using Tissue Quest software (Gnostics, Vienna, Austria).

IHC was also performed to examine possible accumulated FUS-induced effects on neuronal morphology. Tissue sections were stained overnight at 4 °C with primary antibodies, including neuronal nuclear antigen (NeuN) and anti-neuron-specific class III beta-tubulin (TuJ1) antibodies. After rinsing in phosphate-buffered saline, the sections were incubated in secondary antibody with goat anti-rabbit fluorescence 594 or donkey anti-mouse fluorescence 594 for 1 hour at room temperature. After rinsing in PBS, coverslips were applied to slides with anti-fade reagent and the nuclear marker DAPI (4′,6-diaminino-2-phenylindole). Finally, the sections were imaged by a Leica TCS SP2 confocal microscope (Leica Microsystems, Wetzlar, Germany).

### Statistical analysis

Statistical analysis was performed using SPSS software (IBM SPSS statistics; IBM Corp., Armonk, NY, USA). SSEP and fMRI data were analysed by repeated-measures ANOVA with LSD *post-hoc* analysis. Histology data were analysed by one-way ANOVA. The *p*-value for statistical significance was 0.05. Data are presented as mean ± standard error of the mean.

## Additional Information

**How to cite this article**: Chu, P.-C. *et al.* Neuromodulation accompanying focused ultrasound-induced blood–brain barrier opening. *Sci. Rep.* **5**, 15477; doi: 10.1038/srep15477 (2015).

## Supplementary Material

Supplementary Information
