# Supplementary material for: Neuromodulation accompanying focused ultrasound-induced blood-brain barrier opening
Source: Sci Rep. 2015 Oct 22;5:15477. doi: 10.1038/srep15477 (PMC4614673; doi:10.1038/srep15477)
Supplement: Supplementary Information [file srep15477-s1.doc]

Supplementary Information

**Neuromodulation accompanying focused ultrasound-induced blood-brain barrier opening**

## Po-Chun Chu^1,+^, Hao-Li Liu^1,2+^, Hsin-Yi Lai^3^, Chung-Yin Lin^2^, Hong-Chieh Tsai^4,5^*, & Yu-Cheng Pei^3,4^*

^1^Department of Electrical Engineering, Chang Gung University, 259 Wen-Hwa 1st Road, Kwei-Shan Tao-Yuan, Taiwan, 333, ^2^Medical Imaging Research Center, Institute for Radiological Research, Chang Gung University/Chang Gung Memorial Hospital, Taoyuan, Taiwan 333, ^3^Department of Physical Medicine and Rehabilitation, Chang Gung Memorial Hospital, 5 Fu-shin Street, Kwei-Shan, Tao-Yuan, Taiwan, 333, R.O.C. ^4^School of Medicine, Chang Gung University, 259 Wen-Hwa 1st Road, Kwei-Shan Tao-Yuan, Taiwan, 333, R.O.C. ^5^Department of Neurosurgery, Chang Gung Memorial Hospital, 5 Fu-shin Street, Kwei-Shan, Tao-Yuan, Taiwan, 333, R.O.C.

**^+^ These two authors contributed equal****ly to this work**

***Address correspondence to:**

Yu-Cheng Pei, M.D. Ph.D.

Tel: +886-3-3281200 ext 3846; Fax: +886-3-3281200 ext 2667;

E-mail: [yspeii@gmail.com](mailto:yspeii@gmail.com)

Hong-Chieh Tsai, M.D.

Tel: +886-3-3281200 ext 2412; Fax: +886-3-3285818;

E-mail: [newcomer9999@gmail.com](mailto:newcomer9999@gmail.com)

**Animal experiments summary**

For the SSEP experiments, eight animals were included in each of the control, 0.3-, 0.55- and 0.8-MI groups, and six animals in the 0.8-MI FUS-alone group. For fMRI evaluation, four animal experiments were conducted in each group. For histological evaluation, nine animals were used in each group (Table S1).

**Table S1.** Summary of animal experiments.

| Experiment | Group | Animal number | MI | Microbubbles |
| --- | --- | --- | --- | --- |
| SSEPs | Control | 8 | N/A | + |
|  | 0.8-MI FUS-alone^*^ | 6 | 0.8 | - |
|  | 0.3-MI | 8 | 0.3 | + |
|  | 0.55-MI | 8 | 0.55 | + |
|  | 0.8-MI | 8 | 0.8 | + |
|  | Re-0.55-MI | 6 | 0.55 | + |
| fMRI | Control | 4 | N/A | + |
|  | 0.8-MI FUS-alone^*^ | 4 | 0.8 | - |
|  | 0.3-MI | 4 | 0.3 | + |
|  | 0.55-MI | 4 | 0.55 | + |
|  | 0.8-MI | 4 | 0.8 | + |
| Histology | Control | 9^†^ | N/A | + |
|  | 0.8-MI FUS-alone^*^ | 9^†^ | 0.8 | - |
|  | 0.3-MI | 9^†^ | 0.3 | + |
|  | 0.55-MI | 9^†^ | 0.55 | + |
|  | 0.8-MI | 9^†^ | 0.8 | + |
|  | Re-0.55-MI^‡^ | 9^‡^ | 0.55 | + |

^*^ Results presented in Supplementary Information online. ^†^ Three animals were sacrificed at each of the three post-FUS time points: 1 h, 2 days or 7 days. MI: mechanical index. ^‡^ Three animals for each of the times points (4, 7 and 10 days). Re-0.55-MI: repetitive 0.55-MI FUS-induced BBB opening. IHC = Immunohistochemistry.

**0.8-MI FUS-alone group**

FUS without microbubbles can also induce neuromodulation ^1,2^ so it is important to test whether the FUS we used can alter SSEPs. To this end, we presented FUS with MI level of 0.8 (and no microbubbles), which is the maximum FUS level we used in the BBB-opening experiment. The FUS, Evans blue dye, SSEP, BOLD and HE staining methods were identical to those used for the 0.8-MI group, except for the absence of microbubbles.

**Evans blue dye**

Evans blue dye leakage was not observed in any animals that received 0.8-MI FUS without microbubbles, indicating the absence of BBB opening (Fig. S1A).

**SSEPs**

Reduced P1 amplitude was not observed in the FUS side within the first hour (Fig. S1B (i), repeated-measures ANOVA, *F* (7, 84) = 1.35, *p* = 0.24) and on long-term follow-up post-FUS (Fig. S1B (ii), *F* (3, 33) = 0.57, *p* = 0.64). Similarly, no prolongation of P1 latency was found within the first hour (Fig. S1B (iii), *F* (7, 84) = 0.39, *p* = 0.91) and on long-term follow-up post-FUS (Fig. S1B (iv), *F* (3, 33) = 0.29, *p* = 0.84), indicating that 0.8-MI FUS-alone did not alter SSEPs.

**fMRI**

BOLD responses in left S1FL elicited by forepaw electrical stimulation were not reduced by FUS (*F* (3, 18) = 0.85, *p* = 0.49) (Fig. S1C), a finding that was compatible with results for SSEPs.

**Histology**

0.8-MI FUS-alone induced no RBC extravasations (*p* > 0.05) (Fig. S1D).

**Conclusion**

In summary, the 0.8-MI FUS without microbubbles induced no noticeable neuromodulation as evidenced by no changes in SSEPs, BOLD responses, or histological findings.


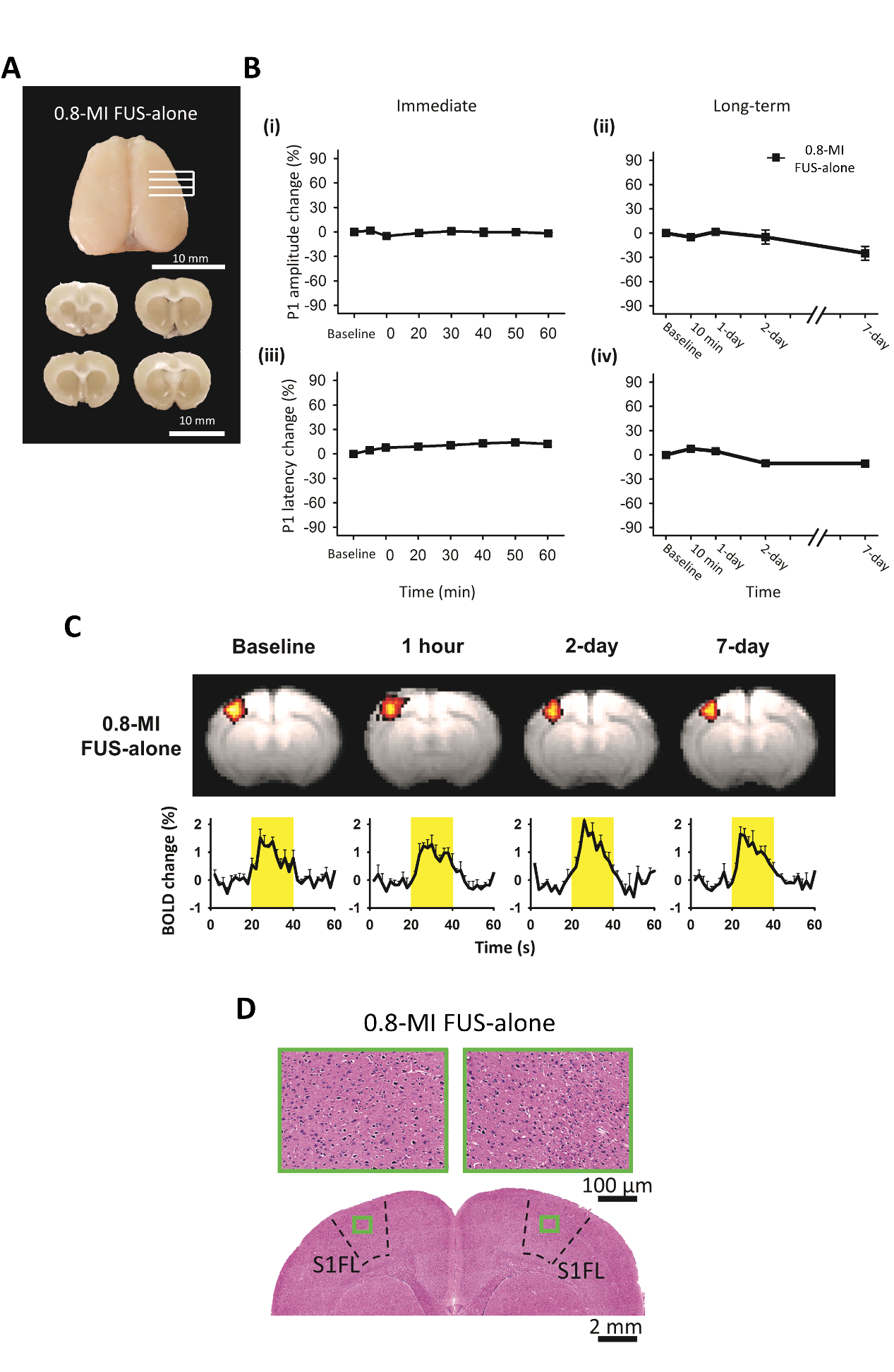


**Figure S1.** Results of 0.8-MI FUS-alone group. (**A**) Representative gross views and brain slices for the 0.8-MI FUS-alone group. Evans blue dye leakage was not found for the 0.8-MI FUS-alone group, indicating no FUS-induced BBB opening. (**B**) The immediate and long-term changes in SSEPs in the left S1FL. (i) Change in P1 amplitude within the first hour post-FUS. (ii) Change in P1 amplitude on long-term follow-up. (iii) Change in P1 latency within the first hour post-FUS. (iv) Change in P1 latency on long-term follow-up. 0.8-MI FUS-alone induced no changes in SSEP amplitude or latency. (**C**) Spread and magnitude of BOLD responses. No neuromodulation effect was observed for the 0.8-MI FUS-alone group. (**D**) Example HE-stained slices 1 h post-FUS. 0.8-MI FUS-alone induced no RBC extravasations.

**SSEP samples on the FUS side**

The immediate effect of FUS exposure within the first hour after exposure was to suppress the P1 amplitude on the FUS side (left) in the 0.55- and 0.8-MI groups, but not in the control, 0.3-MI or 0.8-MI FUS-alone groups (Fig. S2*A*), indicating that SSEP changes only occurred when high MI FUS induced BBB opening.

Long-term follow-up at 2 and 7 days showed that the suppression of P1 amplitude on the FUS side lasted for at least one week in the 0.8-MI group, but not in the other four groups (Fig. S2B).

**
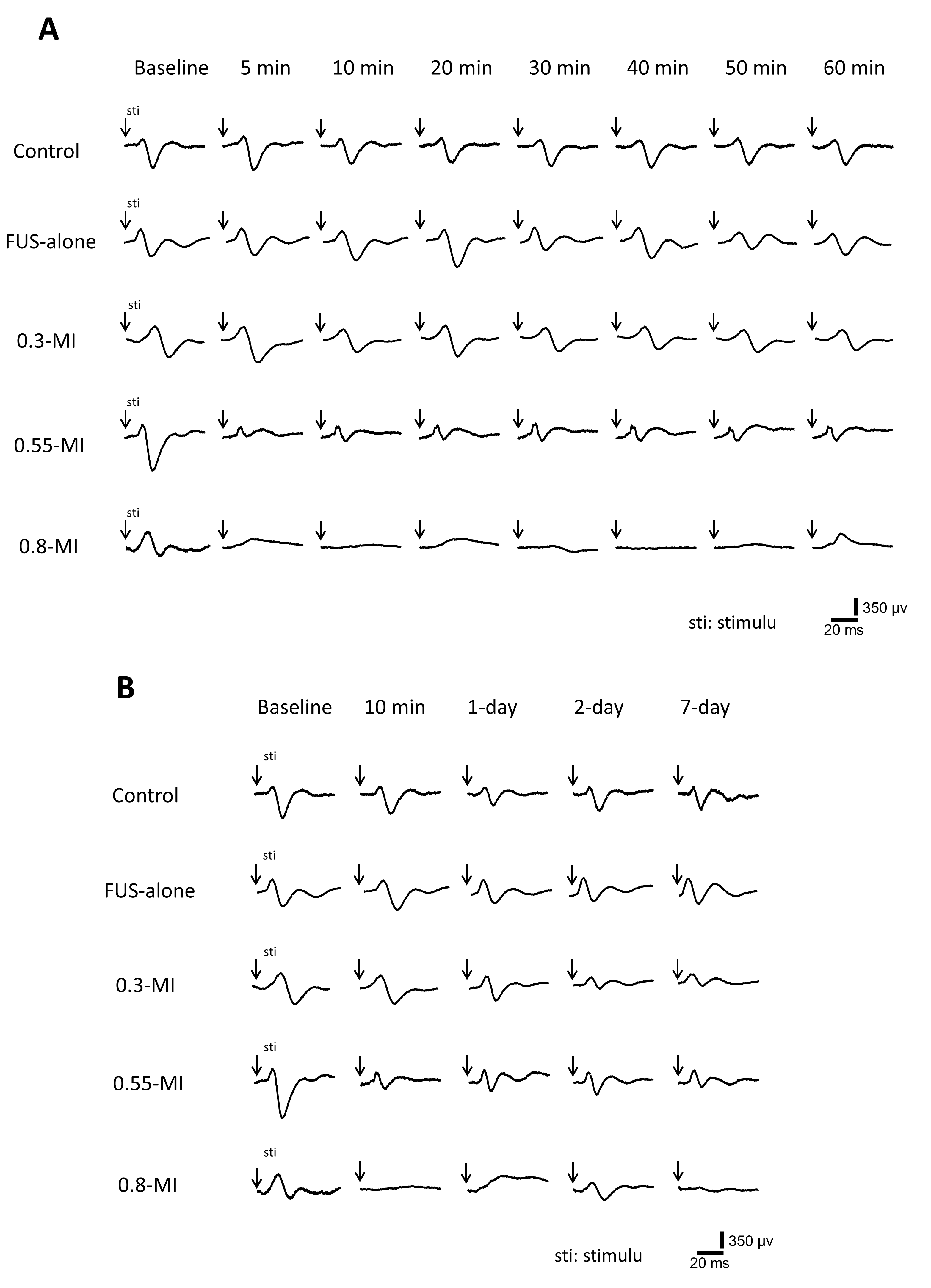
**

**Figure S2.** The immediate and long-term changes in SSEPs in the FUS side. (**A**) Example SSEP traces at the left S1FL within 1 hour following FUS. (**B**) Example SSEP traces at the left S1FL for 1 week following FUS. Significantly altered SSEP traces were observed in the 0.8-MI FUS group, but not in other groups.

**SSEPs recorded on the non-FUS side**

Within the first hour, FUS to the left S1FL did not alter P1 amplitude on the non-FUS (right) side when elicited by left forepaw electrical stimulation (Fig. S3A). (group effect, *F* (3, 28) = 0.243, *p* = 0.87; interaction effect, *F* (18, 168) = 0.47, *p* = 0.98). Follow-up on 2 and 7 days later showed that P1 amplitude was also unaffected on the non-FUS side (Fig. S3B) (group effect, *F* (3, 25) = 0.14 *p* = 0.94; interaction effect, *F* (9, 75) = 0.43, *p* = 0.92).

Within the first hour post-FUS, P1 latency was not prolonged on the non-FUS side (Fig. S3C) (group effect, *F* (3, 28) = 0.29, *p* = 0.83; interaction effect, *F* (18, 168) = 1.78, *p* < 0.05). On long-term follow-up, P1 latency was not prolonged on the non-FUS side (Fig. S3D) (group effect, *F* (3, 25) = 0.62, *p* = 0.61; interaction effect, *F* (9, 75) = 0.92, *p* = 0.45).


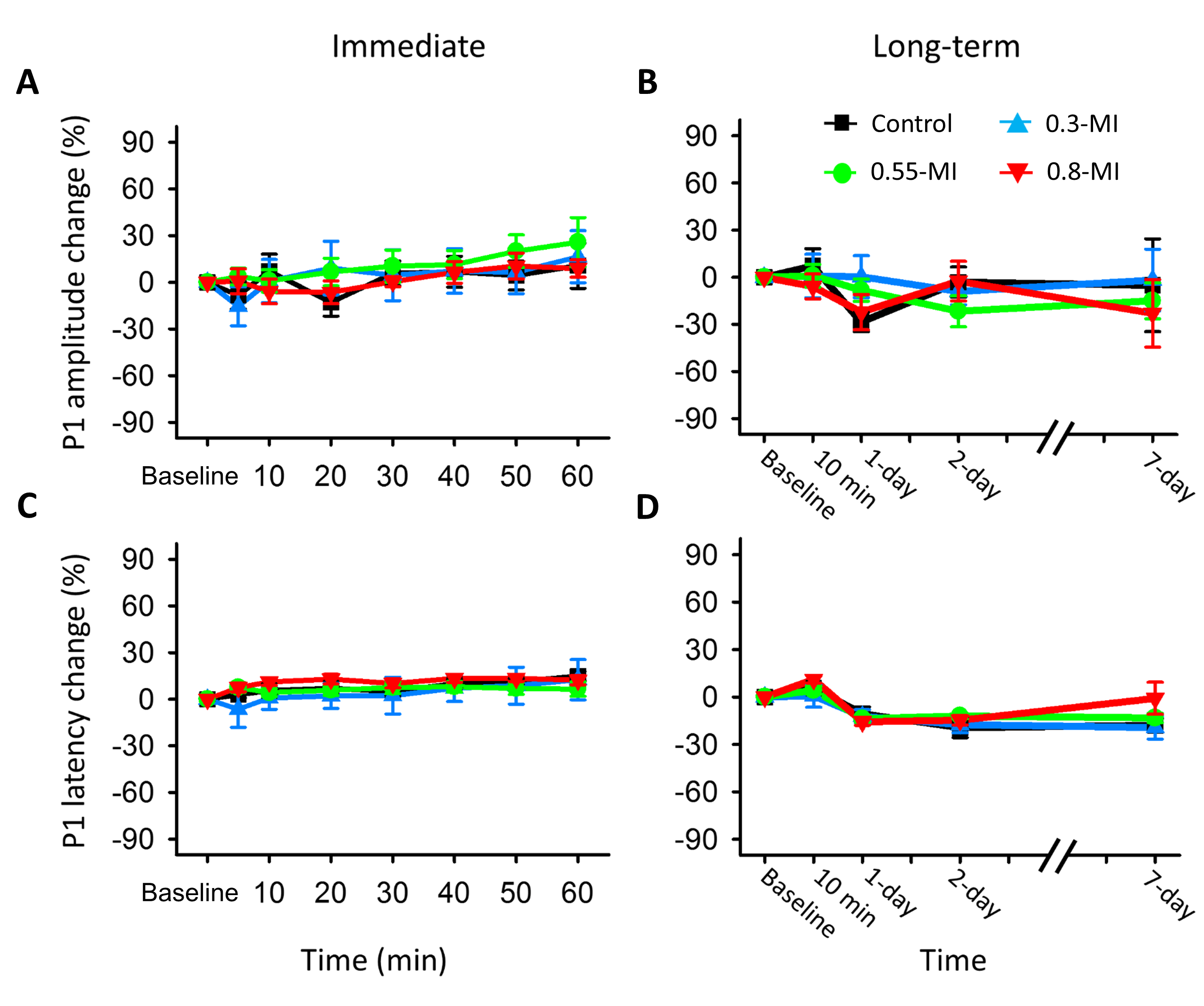


**Figure S3.** The immediate and long-term changes in SSEPs in the non-FUS (right) S1FL. (**A**) Change in P1 amplitude within the first hour post-FUS. (**B**) Change in P1 amplitude on long-term follow-up. (**C**) Change in P1 latency within the first hour post-FUS. (**D**) Change in P1 latency on long-term follow-up. FUS did not alter SSEP amplitude or latency on the non-FUS side.

**Reference**

1 Yoo, S. S. *et al.* Focused ultrasound modulates region-specific brain activity. *NeuroImage* **56**, 1267-1275, doi:10.1016/j.neuroimage.2011.02.058 (2011).

2 Legon, W. *et al.* Transcranial focused ultrasound modulates the activity of primary somatosensory cortex in humans. *Nat Neurosci* **17**, 322-329, doi:10.1038/nn.3620 (2014).
